# INTRACELLULAR WATER CONTENT IN LEAN MASS IS ASSOCIATED WITH MUSCLE FUNCTION AND PHYSICAL ACTIVITY AMONG OLDER ADULTS

**DOI:** 10.1093/geroni/igac059.2594

**Published:** 2022-12-20

**Authors:** Murad Taani, Kimberly Dembinski

**Affiliations:** University of Wisconsin-Milwaukee, Milwaukee, Wisconsin, United States; Ovation Communities, Milwaukee, Wisconsin, United States

## Abstract

High intracellular water (ICW) content in lean mass (LM) has been associated with better functional capacity and lower frailty risk in older adults and suggested to be a useful muscle quality indicator. However, limited research exists on the relationship of ICW to muscle function and physical activity among older adults. The purpose of this study is to examine the relationship between ICW/LM ratio and muscle function and physical activity among older adults. In a cross-sectional correlational study of older adults aged 70 years or older, a tetrapolar bioimpedance spectroscopy device (Impedimed SFB7, Australia) was used to assess both ICW and LM, and the ICW/LM ratio (mL/kg) was calculated. The Short Physical Performance Battery (SPPB) and Timed Up and Go (TUG) tests were used to measure muscle function. The Actigraph GT3X+ accelerometer (Actigraph, Inc., FL, USA) was used to measure light and moderate-vigorous physical activity and steps per day. Multiple regression analyses were used. For 96 recruited participants (mean age 82.5 ± 7.4 years; 79 [82.3%] were female), mean ICW/LM ratio was 245.1 ± 28.1 mL/kg. The ICW/LM ratio was positively associated with SPPB score, gait speed, moderate-vigorous physical activity, and steps per day, after adjusting for age, sex, and LM. The ICW/LM ratio was negatively associated with TUG score, after adjusting for age, sex, and LM. The findings suggest that intracellular water content in lean mass may affect muscle function and physical activity among older adults. Further studies are needed to verify these findings and determine any potential cause-and-effect relationships.

